# Editorial: Scarcity, regulation, and the abundance society

**DOI:** 10.3389/frma.2022.1104460

**Published:** 2023-01-25

**Authors:** Deven R. Desai, Mark A. Lemley

**Affiliations:** ^1^Georgia Institute of Technology, Scheller College of Business, Atlanta, GA, United States; ^2^Stanford Law School, Stanford, CA, United States; ^3^Lex-Lumina PLLC, Los Altos, CA, United States

**Keywords:** intellectual property, scarcity and abundance, regulating access and benefit sharing, abundance, Internet

New technologies continue to democratize, decentralize, and disrupt production, offering the possibility that scarcity will be a thing of the past for many industries. We call these technologies of abundance. But our economy and our legal institutions are based on scarcity.

Abundance lowers costs. When that happens, the elimination of scarcity changes the economics of how goods and services are produced and distributed. This doesn't just follow a normal demand curve pattern—consumption increases as price declines. Rather, special things happen when costs approach zero.

Digitization and its effects on the production, organization, and distribution of information provide early examples of changes to markets and industries. Copyright industries went through upheaval and demands for new protections. But they are not alone. New technologies such as 3D printing, Cas-9 Cripsr, artificial intelligence, synthetic biology, and more are democratizing, decentralizing, and disrupting production in food and alcohol production, biotechnologies, and more, and even the production of innovation itself, opening the prospect of an abundance society in which people can print or otherwise obtain the things they want, including living organisms, on-demand.

Abundance changes the social as well as economic context of markets. How will markets and legal institutions based on scarcity react when it is gone? Will we try to replicate that scarcity by imposing legal rules, as IP law does? Will the abundance of some things just create new forms of scarcity in others—the raw materials that feed 3D printers, for instance, or the electricity needed to feed AIs and cryptocurrency? Will we come up with new forms of artificial scarcity, as brands and non-fungible tokens (NFTs) do? Or will we reorder our economics and our society to focus on things other than scarcity? If so, what will that look like? And how will abundance affect the distribution of resources in society? Will we reverse the long-standing trend toward greater income inequality? Or will society find new ways to distinguish the haves from the have-nots?

Society already has examples of each type of response. The copyright industries survived the end of scarcity, and indeed thrived, not by turning to the law but by changing business practices, leveraging the scarcity inherent to live performances and using streaming technology to remove the market structures that fed unauthorized copying, and by reorganizing around distribution networks rather than content creators. Newsgathering, reporting, and distribution face challenges flowing from democratized, decentralized, and disrupted production. Luxury brands and NFTs offer examples of artificial scarcity created to reinforce a sort of modern sumptuary code. And we have seen effective, decentralized production based on economics of abundance in examples ranging from open-source software to Wikipedia.

In this introductory essay, we survey the potential futures of a post-scarcity society and offer some thoughts as to more (and less) socially productive ways to respond to the death of scarcity.

## Beyond the economics of scarcity

### Information, digitization, and scarcity

#### Information goods and the success of abundance

Questions about scarcity and abundance are central to how humans organize societies. Traditional capitalist economics is based on scarcity (Frischmann and Lemley, 2007). Things are valuable because they are scarce. The more abundant they become, the cheaper they become. We pay for things because it takes resources—land, raw materials, human labor—to produce them. In general, the more resources it takes to produce them, the more we pay (Samuelson and Nordhaus, 2010). The most fundamental graph in economics shows a supply curve and a demand curve. The supply curve slopes up because resources are scarce, and the demand curve slopes down because money too is scarce. Generally speaking, markets meet in the middle—when it costs more to make something than people are willing to pay for it, manufacturers stop making it. When there are exceptions—when customers are willing to pay a great deal for something that is cheap to make—the producer may make a substantial profit in the short term. But in the long run, other producers, attracted by the high profit margin, enter and offer the cheap product at a lower price, competing away the extra profit margin. Price settles at marginal cost.^1^ Indeed, economics as traditionally taught is the study of how people and society allocate scare resources (Robbins, 2007; Ghosh). When tangible, and often consumable, things such as food, oil, lumber, clothing, are in limited supply, economics tries to explain how to allocate scarce items.^2^ Even if one doesn't consume an item, often only one person can possess it (Frischmann and Lemley, 2007). And in the rare circumstances where that is not true, we often see that as a reason for the government to intervene to provide the good.

The traditional economic story of information is somewhat different. Information is a public good; that is, “one that is non-rivalrous and difficult to exclude non-payers from using” (Wu, 2017; Menell et al., 2022). Unlike, say, ice cream, my consuming information doesn't prevent you from also consuming it. Accordingly, the marginal cost of producing the next copy of information approaches zero (though the physical goods in which information has traditionally been encapsulated, such as books or films, do cost money to produce and distribute). As such, economists worry that things—goods or information—that cost a lot to develop but little or nothing to copy will be underproduced because the ease of copying means producers won't be able to charge enough to recoup their investment in making the thing in the first place (Scherer and Ross, 1990; Landes and Posner, 2003).

For most public goods, the traditional solution is to regulate market entry, designating one company as the exclusive provider of, say, electric power or telephone or cable service, for a particular region and allowing that company to make up its fixed costs by charging its captive customers a price above marginal cost (Samuelson and Nordhaus, 2010). Intellectual Property (IP) laws take a similar approach, creating a right to exclude competition in a particular piece of information so that the creator can make up its fixed costs by charging customers a price above marginal cost (Lemley, 1997; Boyle, 2009).^3^ Unlike more traditional regulated industries, however, the government does not regulate the price IP owners can charge, but instead relies on some combination of the temporary duration of the IP right and imperfect competition from other inventions to keep prices in line (Abramowicz, 2004; Yoo, 2006, 2009; Lemley and McKenna, 2011).

In effect, the point of IP laws is to take a public good that is naturally non-rivalrous and make it artificially scarce, allowing the owner to control how many copies of the good can be made and at what price. In so doing, IP tries to fit information into the traditional economic theory of goods. The fit is imperfect, though, both because IP's restriction on competition creates a deadweight loss to consumers who would have bought the good at a lower price and because the very existence of the IP right means that competition cannot discipline pricing in the same way it does for goods.

But a series of technological changes is underway that promises to end scarcity as we know it for a wide variety of goods. The Internet and related, complementary technologies are the most obvious examples, because the changes flowing from them are furthest along. Even before those changes, the copyright industry offers an earlier example of the way abundance can alter a market to increase rather decrease revenues. The home movie market started as a high-priced one for those who could afford both expensive home video players *and* expensive tapes of movies. Then new technology fostered abundance in the market. First, the machines evolved with VHS winning the format battle. Second, many producers entered the VCR market, and the cost of the machines dropped. Third, people began to buy VCRs to record TV broadcasts. Increased VCR ownership created the opportunity for consumers to buy or rent films on videotape. Following the playbook about costs to copy and the desire for artificial scarcity, studios sought “total control of the cassette from the manufacturer to the customer.” Studios began by pricing copies at $80–$90, so that it made more sense for a rental store to buy and recoup costs with each rental, rather than a home consumer buying a copy. Nonetheless, a few studios experimented with the new market and priced tapes for $19.95 so that more people could own a copy and watch it as often as they liked. By 1996 the rental market was at $9.2 billion and the ownership market was at $7.2 billion with more growth in direct-to-video movies to come (Roehl and Varian, 2001).

These experiments should have told copyright incumbents in music that lowering prices to make illegal copies a less attractive option was the best move. Anti-copying laws and technical measures played their part in the home video market, and technology that hindered getting a clean copy of a recently rented movie likely helped the industry. But that alone was not enough. The combination of a reasonable price point and the fact that street or illegal copies were lower quality allowed a new market and revenue stream to flourish. Although VHS was an analog example of scale and market issues, the lessons carried over with greater force once a series of technological changes reached the industry.

The music industry's experience fighting, and then acquiescing to, digital content is well-known, but tracing the intersection of technologies that led it there shows why more and more sectors could move to a low or post-scarcity equilibrium. The digitization of music was one key change. Physical copies went away in favor of files. Given the low-speed and bandwidth of modem connections, fears of copying were more about digital audio tapes rather than copying digital files and sharing them. The dream of a celestial jukebox was just a dream. But music compression improved. The Internet became commercial. Bandwidth and connection speeds increased. All these complementary technologies converged and unleashed the power to distribute recordings at will for essentially low to no cost.

In addition, software changed the way music was recorded and gave creators access to high-end production techniques. Rather than needing expensive access to recording studios for an adequate demo tape that artists hoped would lead to a recording contract, access to high-end studios, and music producer expertise, artists could make high-quality recordings with high-end production techniques. The cluster of production and distribution technologies democratized and decentralized the music industry.

Digitization is a core, first step toward ending scarcity because it helps remove physical limits. That shift often means producers must adapt to the realities of low-cost copying and distribution acute. Digitization not only affects the way copyrighted products are consumed but also the way they are produced, and thus the nature of the industry in general. Once digitization takes hold of an information market, it dramatically reduces the cost of producing that content. Add in the nature of the Internet and not only does production cost drop, but also other aspects of the market that limit abundance. The Internet accelerates the changes because it reduces the cost of reproduction and distribution of informational content effectively to zero. Furthermore, as the Internet has fostered an abundance of low-cost information creation and sharing, it has created a variety of intermediaries such as search engines and Web hosts that enable access to information for free or at a very low cost. Those intermediaries are agnostic about (and quite often ignorant of) the content they are distributing. In short, digitization and the Internet has disaggregated creation and distribution. I can create without distributing secure in the knowledge that my works will be disseminated by others who distribute without creating.

The result has been a resounding success story. People are creating and distributing more content now than ever before, by at least an order of magnitude (Rifkin, 2014; Lemley, 2015). Economic scholarship suggests that although until around 2011–2013, recording industry revenues have declined substantially from their high in 1999, there were more songs being released than ever before, more new artists than ever before, and more purchases of music than ever before, and the songs released seem to be of at least as high quality as before the digital disruption of the industry (Lunney, 2012; Waldfogel, 2012).

The claim that music (or video, or text) would stop being produced without the economics of scarcity was proven false (Cohen, 2011; Lemley, 2011). But that doesn't mean digital technologies brought no disruption. Incumbents had to retool their business models. High-cost intermediaries and distribution networks changed or went out of business. A world of four or five major labels controlling close to 80 percent of the market shifted, and a host of smaller labels produced more music. Artists sold their work directly to consumers. Apple's iTunes, Amazon, and GooglePlay began selling singles at 99 cents to a dollar 30 cents. Rhapsody and Spotify developed subscription services. Concerts became a major source of income. After some legal fights, YouTube came up with a system to allow rights holders to identify potentially infringing works, and to offer rights holders ways to make money for uses previously too expensive to negotiate even through rights collectives such as the American Society of Composers, Authors, and Publishers (ASCAP) or BMI.

Digitization and network technology shifted the way music is created, sold, and monetized. The practice was democratized. Yet, as one music industry report shows, the industry has experienced 7 years of growth between 2014 and 2021, with 2021 global revenues totaling $25.9 billion, an 18.5 percent increase from 2020 (Richter, 2022). Perhaps counter-intuitively, the bottom was in 2014, the year streaming began; and it was the advent and embrace of streaming that returned the market to growth. Once again technology increased abundance, and the industry adapted to that change.

Something similar happened with video, books, and even news reporting. The rise of sites like YouTube has led to an astonishing outpouring of videos from outside Hollywood. More than a decade ago, YouTube had more content added every month than the major TV networks created in 60 years. Since then, the numbers of hours uploaded has grown from 300 to more than 500 h of new content uploaded to YouTube every *minute*. At the same time, despite the COVID pandemic's effect on movie theater attendance, the movie industry is faring better than ever before in history (McClintock, 2021). This success is in part because of the industry's embrace of streaming content, a technology that seemed to threaten the industry a decade ago (Oberholzer-Gee and Strumpf, 2010). People are buying more books than ever before, with print books still accounting for 76 percent of sales revenues in 2021.^4^ And while the price of those books has declined somewhat, writers are also publishing more books than ever before, including a surprising number of successful self-published books (Oberholzer-Gee and Strumpf, 2010; Waldfogel and Reimers, 2015). Print newspapers have seen revenues decline because of the Internet (Edmonds, 2012), but that doesn't mean news reporting has declined; more news is reported more quickly from more sources as individual citizens are increasingly capable of documenting the world around them. Nor has the quality of journalism necessarily fallen; indeed, one recent study finds that “newspaper content appears to be getting more sophisticated in response to increased Internet penetration” (Salami and Seamans, 2014). True, there is lots of misinformation out there, and that's a problem. But there is also lots more factual news reporting than in prior eras. And despite piracy, both the film and publishing industries reported higher profit margins in the 2010s than they did a decade before (Band and Gerafi, 2013). Live music and shows have also reached unprecedented levels of revenue and profit. Overall, the picture of the entertainment industry is far from bleak; the overall industry grew from $449 billion in 1998 to $745 billion in 2010 (Travis, 2015).

Perhaps most surprising, people are creating an astonishing array of content specifically for the purpose of giving it away for free on the Internet. Early on, scholars worried that no one would create content for the Internet because they couldn't see a way to get paid (Ginsburg, 1995), but it is hard to think of a prediction in all of history that has been more dramatically wrong. People spend hundreds of millions—or even billions—of hours a year creating content online for no reason other than to share it with the world. They create and edit Wikipedia pages, post favorite recipes, create guides to TV shows and video games, review stores and restaurants, and post information on any subject you can imagine (Benkler, 2002, 2006; Rimmer, 2009). The claim that people would not create *and share their creations* because of the public goods aspect of information, as the economics of scarcity predicts, has not been borne out. Rather, even in the analog days, we all knew of garage bands, artists, tinkers, and other creators whose worked was local and under the radar. The shift to digital, networked creation has unearthed these creative efforts and provided new ways to share them. If, as Doctor Johnson famously suggested, “[n]o man but a blockhead ever wrote except for money,” Johnson (1884) we are a world of blockheads, gleefully creating and sharing all sorts of content with the world. Ghosh's and Asay's contributions to this volume note the fundamental nature of the changes the Internet has wrought on copyright and incentives to create (Ghosh; Asay) and Said discusses how copyright law uses the rhetoric of scarcity to justify its continued dominion.

#### Digitizing physical goods: The promise of abundance

More recently, new technologies promise to do for a variety of physical goods and even services what digitization and the Internet has already done for information. 3D printers can manufacture physical goods based on any digital design (Desai and Magliocca, 2014; Newcomb, 2022). But that has been the case for a range of computer-numeric-control devices for some time. The difference is the intersection of increasingly sophisticated yet lower cost 3D printers; ever more accurate and inexpensive scanners; and leaps in material science allowing 3D printers to move beyond plastics to cement, ceramics, metals, and more. Together these changes have spawned an abundance of the know-how and means to produce things that were once the province of high-cost manufacturing firms in industries as varied as toys, guns, autos, homes, drugs, and even spaceships. China is even pursuing building an entire hydro-electric dam using 3D printing, robots, and artificial intelligence systems, but almost no humans. Several industries use versions of this technology to make better prototypes and bring new products to market faster, but something else is happening too. New players are entering industries, such as the car industry, where start-up costs used to be high and acted as a barrier to entry.

For example, Local Motors was able to use crowd sourcing to design a car with the winning designer receiving $7,500, and then complete the prototype in a little over 2 months. The two-seater has only 49 parts, most of which were made with 3D printing technology. The third production of the prototype took about 40 h to build. The body itself is a one-piece carbon tub. One car reviewer noted that the other car he tested with a one-piece carbon tub body was a McLaren 650S priced at more than $300,000. Local Motors plans on releasing its first vehicle sometime in 2016 at price between $18,000 and $30,000. In addition, the approach of Local Motors allows it to build mini-factories for far less than the billion or so dollars traditional carmakers such as Tesla spend (yes Tesla is traditional on this point). That means Local Motors should have been able to adapt faster, deliver closer to consumers, and offer custom, high-quality, low-cost cars.^5^

The amount of high-end technology bought to market at low-cost shows that the ability to tinker and create even in a complex sector such as the automotive industry is real and persists. For example, in 2019, BMW revealed a 3D printable concept car, yet a father and son had already used 3D printing and related CNC technologies to make a Lamborghini at home at a cost of $20,000 investment (Voulpiotis, 2019). Like the Local Motors compared to McLaren, a Aventador Lamborghini on which the 3D printed version is based, cost more than $300,000 (Voulpiotis, 2019). As in other industries facing abundance technologies, incumbents may go after 3D printer sites offering digital plans for parts because of claimed trademark issues (Stumpf, 2022). Or companies may follow the lead of GE Aviation, Lockheed Martin, Raytheon, Honeywell, and Siemens Energy, that have agreed to work on changing their supply chain by supporting U.S. companies embrace 3D printing and similar technologies—a move that fits with the Biden Administration's Additive Manufacturing Forward program (Shabad, 2022).

In other markets, consumers and tinkerers are creating and sharing plans for homemade toys and even guns. Some of these creations are new, and some build on offerings already in the marketplace. Like the copyright industry, industries that rely on patents are seeing small industry and individuals “interact” with their IP much more than was possible just a decade ago. Both Matthew Rimmer and Shane Greenstein provide additional examples in their chapters in this volume. Rimmer discusses the development of metal 3D printing and how it is changing manufacturing, while Greenstein discusses how print on demand clothing is changing the nature of fast fashion.

Synthetic biology has automated the manufacture of copies of not just existing genetic sequences, but also any custom-made gene sequence, allowing anyone who wants to create a gene sequence of their own to upload the sequence to a company that will “print” it using the basic building blocks of genetics. In addition, two related technologies, CRISPR and Cas9, have lowered the bar to genetic editing. CRISPR stands for “Clustered regularly-interspaced short palindromic repeats [which] are segments of bacterial DNA that, when paired with a specific guide protein, such as Cas9 (CRISPR associated protein 9), can be used to make targeted cuts in an organism's genome.” (National Academies of Sciences, 2016). Because of CRISPR/Cas9, gene editing has gone from being “laborious and time-consuming” Kreiger (2016) to being “facile and rapidly achievable” (Sternberg and Doudna, 2015). At least one scientist now offers a DIY gene-editing system that is a simplified version of CRISPR for $120, and he offers “lab protocols, inexpensive equipment, and tutorials” so that the general public can learn the basics of gene editing (Sternberg and Doudna, 2015). The democratization of genetic science is in full-swing.

Advances in robotics and AI generalize the principle beyond goods, offering the prospect that many of the services humans now supply will be provided free of charge by general-purpose machines that can be programmed to perform a variety of complex functions (Lemley and Casey, 2019; Greene, 2022).

While these technologies are not nearly as far along as music and film, the changes in these industries share two essential characteristics with technology's influence on music and film: The technological advances radically reduce the cost of production and distribution of things, and they separate the informational content of those things (the design) from their manufacture. That latter characteristic is critical, because it means that technologies that once required individual physical investment with specific materials, labor, and plants can now be produced with generic technology. Sometimes that generic technology is nothing more than a computer. But even if it requires manufacturing, computer-aided design and manufacturing mean that a wide array of things can be made with off-the-shelf materials. Combine these technological developments—the Internet, 3D printing, robotics, and synthetic biology—and it is entirely plausible to envision a not-too-distant world in which most things that people want in a wide array of fields can be downloaded and created on site for very little money—essentially the cost of raw materials. Perhaps more important given recent changes in supply chains—be they from COVID'S effect on where, how, and when people worked; new demands for green transportation; or the Russia-Ukraine War's effect on fuel and grain supplies—is the promise of distributed, on-site manufacturing.^6^ Jeremy Rifkin calls this the “zero marginal cost society” (Rifkin, 2014).

If we can avoid the dystopian future of technologically-backed lockdown, the future of many forms of creation is likely to follow the patterns of digitization, decentralization, and democratization. In some cases, such as with things covered by copyright, incumbent industries may embrace the news forms of creation and distribution such as what happened with streaming, while many other creators might leverage copyright to license works depending on whether the creator wants credit, income, or the way a licensor wishes to use the work. Yet the number of people on TikTok alone shows that millions of people are creating and sharing copyrighted works for a range of reasons.

Beyond copyright, lots of people will create lots of designs, code, and biobricks that will enable us to use new production technologies to create more physical things. Other people will use, repurpose, and improve on those things, often without paying. But people will continue to create, because some people will pay for their creations, because there will be other ways to make money from being creative, because they want to be known for something or want the feeling of accomplishment that comes with creating, and, ultimately, simply because they can. In some cases, creators use IP to enable sharing and require attribution credit in non-commercial contexts^7^ while maintaining rights to charge license fees in commercial contexts (Doctorow, 2006). As one example, Cory Doctorow explicitly gives away his novels and lets people use them in one medium and sells them as bound books as well because his overall goal is to be found. As he puts it, his evangelical fans don't “just sell books—[they] sell[] me” (Doctorow, 2006). His fame and his presence leads to paying opportunities because he is the scare resource. As he says, “I've been giving away my books ever since my first novel came out, and boy has it ever made me a bunch of money” (Doctorow, 2006). Yet, more and more of these creations will operate outside the IP system, either expressly (biobrick inventors who choose not to patent their inventions, for instance) or by the simple virtue of ignoring that system.^8^

This future is not a utopia. None of these technologies is perfect, and each requires physical inputs that will in turn be subject to the laws of scarcity. Further, the lesson of digitization and the Internet is that while cheap, democratized production drives more creation, not less, it may also change the nature of that creation. Without IP rights we may see more creation by amateurs and academics and less by professional creators, just as in music we now see more new bands and fewer bands with multi-album staying power. That is both a good and a bad thing; removing the requirement of a major label record contract has surfaced new talent and enabled it to enter the music market, but the decline of professional artists may change the nature of music in ways that cause us to lose some music we'd like to have. Similarly, it is possible to imagine both a wealth of new product designs for 3D printers and a decline in the number of professional design firms. And in synthetic biology and genetics, where at least some products, like viruses and FDA-controlled chemicals, are likely to be heavily regulated, the cost and delay associated with that regulation may require some means to recoup investment.

At least in the medium term, however, professional firms are likely to coexist with the amateurs, just as professional musicians and movie studios have found it possible to coexist—even thrive—alongside the new entrants. The dramatic reduction in cost that has spurred new entry also boosted the demand for content—people consume more music and video content than ever before, for example—and people are willing to pay for things they like if they are delivered in convenient packages. And IP rights are unlikely to disappear even if they are increasingly flouted, so professional providers who choose to rely on IP rather than sharing their work for free can still make some money by doing so.^9^

In short, the technologies of abundance offer a world in which people create more things at less cost, largely despite rather than because of IP laws. IP laws will continue to exist, and they will provide a necessary incentive for some forms of creativity. But creation that relies on IP is likely to play a less and less significant role in a post-scarcity world.

### What remains: Transforming the physical

We come to the scarcity-abundance tension from intellectual property (IP) and information law perspectives, but we acknowledge that not everything can be digitized (Desai, 2014; Desai and Magliocca, 2014; Lemley, 2015). Many things still need to be made and delivered. An abundance society still requires the production of raw materials and infrastructure—food, energy, and the feedstock for 3D printers, data centers, communications infrastructure, and so on. As the population grows, the demand for more food and energy persists. And the response to prior technologies of abundance in capitalist societies has been to demand more stuff, increasing production and consumption. One possibility is that we start the cycle of consumption all over again.^10^ But even in non-information fields technologies of abundance may change the landscape.

Agriculture offers a perspective on the interplay of technology and abundance. As one report sums up, despite a population boom between 1900 and 2011, Malthusian fears of starvation did not materialize. Instead, the world went from 1.7 billion to 7 billion people while still “produc[in] enough calories in 2012 to feed the entire population, plus an additional 1.6 billion people” (Johns Hopkins Center for a Livable Future, 2022). Advances in food production technology such the development of fertilizers or the genetic engineering behind the Green Revolution allowed greater yields. Other changes such as tractors and harvesters reduced the amount of human and animal labor needed to farm and the efficiency of a given farm plot (Dimitri et al., 2005; Johns Hopkins Center for a Livable Future, 2022). The invention of refrigeration allowed crops to be grown in lush farmlands and shipped to urban centers across the U.S. and the world. These changes increased food security such that India—a country with hundreds of millions of mouths to feed—became a net exporter. In sum, several technologies—shared and improved food stock such as corn, rice, sweet potatoes, and cassava; transportation innovation in rail and shipping; new methods for storing food in larger amounts and over long distances; and synthetic fertilizers—converged to create abundance.

The history of agriculture in the U.S. shows more about the way technologies of abundance alter a sector and society. There was a time when over 60% of the people in the United States were primarily employed producing food (Rifkin, 2014).^11^ Even in 1900 the number was 41% (Dimitri et al., 2005). The dropoff continued such that by 1930 the number was 21.5%; by 1945 16%; by 1970 4%; until by 2002 the number was below 2% (Dimitri et al., 2005). Comparing two other metrics shows where technologies of abundance led to major shifts in how we live and work. Agricultural GDP was 7.7% of total GDP in 1930; 6.8% in 1945; 2.3% in 1970; and 0.7% in 2002 (Dimitri et al., 2005). Mechanization changed farming as well. In 1900, 21.6 million work animals were used in farming. By 1930 the Census reported 18.7 million horses and mules and 920,000 tractors in use; by 1945, 11.6 million horses and mules and 2.4 million tractors; by 1960, 3 million horses and mules and 4.7 million tractors (the Census stopped keeping this data in 1960; Dimitri et al., 2005). As farms embraced technology that improved production, the amount for human labor needed of course went down. Thus, both food and labor moved from scarcity to abundance. Those changes were dramatic, more dramatic than anything we face today.

What would people do when they no longer needed to grow food to survive? The answer is instructive: They would do a whole array of things no one in 1800 had ever imagined, often simply because they could. They were freed from the need grow their own food and turned loose to create new things and new means of passing their time. This wasn't all leisure time, of course, though Americans in the twentieth century worked many fewer hours than in the nineteenth century. But even working to put food on the table no longer meant growing that food for most. They could make and do other things and use some of the money they earned to buy food from the dwindling number of farmers. The abundance of labor and time contributed to the Industrial Revolution (Overton, 1996), which brought dramatic change of its own but also unprecedented improvement in the human condition.

Today we can envision the global equivalent of what happened in the United States over the past 200 years. What becomes possible once we no longer must compete for food? Can we reach a stage of production where human labor and environmental costs are so low that we can provide nutritious food to all? It seems we have enough calories to go around and then some.^12^ Nonetheless, what the U.N. calls prevalence of undernourishment (PoU) exists for 770 million people or almost 10% of the world with continents such as Africa reaching 21% (FAO, 2021). A related issue is food insecurity (lack of access to nutritious and sufficient food, which in 2020 affected “Nearly one in three people in the world (2.37 billion)” (FAO, 2021). The issues are not primarily about abundance but instead access to it.

The problem of having enough food but the food not reaching everyone returns us to scarcity. Food is abundant. Scarcity is social, economic, and political. Recent disruptions to supply because of the COVID Pandemic, extreme weather, and the war in Ukraine increase the barriers to food distribution (Egan, 2022). Volatile food prices and severe food shortages can set off conflicts and increase socio-political unrest (Brück and d'Errico, 2019). As the U.S. Secretary of the Agriculture Tom Vilsack has said, food security allows for a stable democracy (Vilsack, 2022). He also said, “Show me a nation that doesn't feed its people, and I'll show a nation that's looking to try and expand its borders,” as he tied the war in Ukraine to Russia's desire to take over Ukraine's tremendous agricultural output (Vilsack, 2022). If society can reduce or eliminate global food insecurity, not only would people have access to sufficient food but the risk of violent, destabilizing events that damage infrastructure and displace populations should go down.^13^

Producing more food with less effort and having that food reach everyone is thus not the only goal. Even with the today's abundance, concerns about how well current methods are sustainable abound. The farming methods that have created surpluses also create serious negative externalities related to using fossil fuels, unsustainable water management, monoculture farming, the effects of fertilizers and pesticides on soil, and soil erosion (McKenzie, 2007). In addition, the ongoing catastrophe of climate change demands farming techniques that rely less on burning carbon and using fertilizers while maintaining nutrition and increasing yields. These new demands are spurring farming innovations in vertical farming and GMOs that may even shift farming of crops such as tomatoes and strawberries from alternating hemispheres to year-round production in the United States thus increasing access to unprocessed foods and reducing the need to import fruits and vegetables from Central and South America during winter and spring. As technology improves how and where we farm, abundant food should persist and so it will be up to policy makers to solve distribution problems. Wadhwa's chapter in this volume offers some remarkable examples of how they are doing so.

Energy production presents similar production issues, ones where regulation and infrastructure needs intersect and create challenges for the shift to abundance. The energy sector has gone from highly regulated to deregulated; and yet until recently production barriers have meant that large players maintained control over how homes or small communities produce power. Solar and wind power have been around for a long time, but it has taken the increased demand for renewable energy and government subsidies to allow these technologies to reach economies of scale that allow consumers to use them. The move to renewable energy is in full swing, and it is likely to be accelerated both by world events demonstrating the fragility of fossil fuels and the inexorable reality of climate change. Indeed, we may have reached an inflection point. On March 29, 2022 wind power surpassed coal and nuclear power for a full 24 h as a source of US energy (Storrow, 2022). That was possible because recent investments in wind power means that wind power “has grown from about 2 percent of annual American power generation to more than 9 percent” (Storrow, 2022). And the dramatic decline in solar prices has made it not only feasible but cheaper than fossil fuels even before we take into account the considerable social costs of the latter. Wind and solar energy were only 12% of total U.S. energy used in 2021 (U.S. Energy Information Administration, 2022). But with other renewable or non-carbon sources like hydro and nuclear added in, the share of energy generated from sustainable sources will soon be above 50%, and its growth is only accelerating. Wadhwa's chapter in this volume explains why that trend is effectively unstoppable.

Even though technologies of energy abundance exist, political and structural problems can hinder society's ability to use them well, revealing new chokepoints of scarcity. For example, power plants need power lines to reach consumers, but those lines are not being built because of not-in-my-backyard, rights-of-way issues (Friedman, 2022). These barriers are so significant that not even billionaire Philip Anschutz has been able to connect his Wyoming windfarm that could power to nearly 2 million customers to the Southwestern U.S., which desperately needs that power (Friedman, 2022).

Contrasting Germany's experience with the U.S. one shows that political will is needed for abundance technologies to take hold. In 2011, Germany gave up on its nuclear power plants (which are not renewable but do not put carbon in the atmosphere as fossil fuels do), which accounted for almost 25% of its electricity (Friedman, 2022). Germany had no immediate backup plan and turned to coal and gas plants and imported energy to fill the gap (Friedman, 2022). The difference is that Germany also had a plan of tax incentives and subsidies in place to stimulate the switch to renewables (Friedman, 2022; Wehrman, 2022). Just over a decade after Germany began its program, 54% of German energy consumption comes from renewable energy sources (Friedman, 2022).

Other energy sources such as nuclear power will face opposition from some environmental quarters but could reduce energy costs significantly. Unlike solar and possibly wind power, home nuclear power (fission or fusion) is only a science fiction story of the Back to the Future sort. Put differently, the nature of energy production will likely still require one or a few centralized, large players. Regulation will enter as with other public goods and natural monopolies because a decentralized market for nuclear power is not efficient or at least likely to emerge. But even if it is supplemented with large central plants, the production of power, which centralized throughout the twentieth century, is likely to become increasingly decentralized in the twenty-first century. We could and should end up with a well-functioning hybrid system where a combination of centralized and decentralized power generation offers low-cost, abundant, greener, and resilient power.

Digitization and technologies of abundance won't make supply chains a thing of the past. Even with advanced 3D printing, making physical things requires raw materials, and those raw materials must come from somewhere. But by dramatically reducing and simplifying what things must be moved from place to place, abundance technologies offer the promise of making those supply chains simpler, cheaper, and more environmentally friendly.

## Responses to a world of abundance

### Degrees of freedom

We acknowledge that not everyone shares our view of the upsides of abundance. More content is great, but Brett Frischmann and Michael Madison worry that it leads to scarcity of attention span (Madison et al.). More news sources are great, but Kanuri and Pattabhiramaiah worry that it has hollowed out traditional news media and led to a lower overall quality of information. Efficient delivery of that content by leading players is great, but Burstein worries that concentration in communications may take us back to the days of government regulation of speech through the “fairness doctrine.”

And to be clear, we do not think *everything* will abundant; rather we suggest that many more things will be abundant in ways that matter for the economy and the law. The distinction between information-based, non-rival products and rivalrous products matters. As more and more things can be digitized, the costs to create, produce, and distribute those things will go down and approach zero. Thus, on a long time horizon, one can expect an equilibrium with low-costs and nonetheless high production. But even that isn't a guarantee, because abundance may generate demand that consumes what technology has made available. Consider the high electricity costs in two information production sectors, cryptocurrency mining and AI computing. Bitcoin relies on scarcity of computing to create value. High cycle computing faces scarcity of hardware and the costs of running machines at high volume. Both these digital sector activities are information-based and so could be mistaken for the sorts of abundance that nears zero-cost. Truly computationally intensive acts like mining cryptocurrency are cheap but not free. The ability to engage in those acts cheaply has created a new market for computations that couldn't have been conceived of in a world of computational scarcity, one that increases consumption so much it may render scarce what technology made abundant.

These are legitimate concerns. But they do not suggest to us that abundance is a bad thing. Abundance tends to flow from technology. Technology is ambipotent (Lowrance, 1986). It and its outputs can be used for a range of outcomes. In that sense, the concerns suggest that abundance is an output that can be managed. How that management occurs, and how it affects others, is a function not just of technological advancement but of social context.

More generally, we think technologies of abundance open up the *possibility space* for people and societies. More people have at least the potential to make, acquire, and do things they never could before. Whether that potential will be realized depends on whether and how those technologies indicate a need to restructure social and legal relationships and the will to make such changes. We explore some of those potential restructurings, for good and ill, in the following sections.

### Replicating scarcity—Regulation, IP, status goods, and NFTs

The existing economy of scarcity has some powerful, entrenched interests on its side. It also has a sort of intellectual myopia; we find it hard to envision what economic organization looks like in a world without scarcity. Scarcity may even be hard-wired into our brains, which are used to competing for resources. One likely reaction to the elimination of scarcity is to try to replicate it. In this section, we consider several ways that might happen.

#### Regulation of disruptive technologies

The energy sector shows the potential for abundance. It also shows how strong the desire to recapture scarcity profits is. Even California, unquestionably the leader in green tech and climate change mitigation,^14^ shows how a politics that seeks to foster abundance can be hijacked. In 2006, then Governor Arnold Schwarzenegger's administration championed greener energy and the move to solar power. The combination of technology and social policy has led to California having “1.3 million solar rooftops generating roughly 10,000 megawatts of electricity—enough to power three million homes” (Schwarzengger, 2022).

This abundance ought to be welcome, both because it generates cheaper power and because that power is renewable and is not contributing to climate change. But it wasn't welcome to one important constituent: power companies. Power companies generate power, but they also transmit it. And they need revenue to maintain the grid, much less to harden it for the coming climate catastrophe. As more people, often wealthier people, move off the grid, those still on the grid will face higher costs for their energy, because the power company cannot change the nature of the overall grid. These tensions show ways that abundance on one hand can lead to poorer outcomes for the system as a whole.

Claiming to address this problem, and despite California's professed commitment to clean energy, at the end of December 2021, the state tried to cut “by about 80%” the rate paid for energy created by home renewables and add a new “steep grid access charge[], about $60 a month for a typical solar customer” (Anderson, 2022). This was an effort to return to scarcity and the centralized provision of power with which entrenched incumbents were familiar.^15^ California would still support solar energy, according to this proposal; it would just support large industrial solar farms run by the power companies.

Energy companies may need to adjust rates to maintain the overall grid, and indeed we need to invest in modernizing that grid to handle the move to clean energy (Welton, 2021). But the proposed rule sought to gut the advantages of decentralized, democratized technology in favor the utility companies in a way that would run counter to the benefits of abundance. As with all things environmental, the issue is complicated, but this was first and foremost an effort by utilities to hold onto the centralized model of power production that predates technologies of abundance. This is but one example of what Mark Lemley and Mark McKenna have documented—the effort of incumbents across many markets to try to block disruptive technologies (Lemley and McKenna, 2020).

The tendency to try and recapture a market moving to abundance does not mean abundance is doomed. Rather it shows that varying forces can pull, or at least try to pull, a sector moving to abundance back to scarcity and centralized control. Whether that desire succeeds depends on things beyond the technology that enables abundance. Put differently, while technological change creates the possibility of abundance, ending scarcity can happen only if those technologies are coupled with the political will to replace them.

#### IP rights and artificial scarcity

The role of IP in a world of abundance is both controverted and critically important. IP rights are designed to artificially replicate scarcity where it would not otherwise exist. In its simplest form, IP law takes public goods that would otherwise be available to all and artificially restricts their distribution. It makes ideas scarce because then we can bring them into the economy and charge for them, and economics knows how to deal with scarce things. So on one view—the classical view of IP law—a world in which all the value resides in information is a world in which we need IP everywhere, controlling rights over everything, or no one will get paid to create.

That was the initial response of IP law to abundance technologies, but that response is problematic for a couple of reasons. First, it didn't work. By disaggregating creation, production, and distribution, the abundance technologies democratized access to content. Copyright owners were unable to stop a flood of piracy even with 50,000 lawsuits, a host of new and increasingly draconian laws, and a well-funded public education campaign that starts in elementary school. And even targeting the intermediaries proved futile; among the things you can print with a 3D printer is another 3D printer (Orsini, 2014). The world of democratized, disaggregated production may simply not be well-suited to the creation of artificial scarcity through law.^16^

Second, even if we could use IP to rein in all this low-cost production and distribution of stuff, we shouldn't want to. The rationale for patent, copyright, and trade secret has always been not to raise prices and reduce consumption for its own sake, but to encourage people to create things when they otherwise wouldn't. More and more evidence casts doubt on the link between IP and creation, however. Empirical evidence suggests that offering money may actually stifle rather than encourage creativity among individuals. Economic evidence suggests that quite often it is competition, and not the lure of monopoly, that drives corporate innovation (Arrow, 1962). Digitization combined with Internet distribution may have spawned unprecedented piracy, but it has also given rise to the creation of more works of all types than ever before in history, often by several orders of magnitude. Perhaps, as we suggested above, the a series of digital technologies has so reduced the cost of creation that more people will create even without an obvious way to get paid. Or perhaps they never needed the motivation of money, just the ability to create and distribute content. Either way, if the goal of IP is to encourage the creation of new works, the examples of technology driven changes in several IP-based industries suggests that for an increasingly important range of creative works, radically reducing the cost of production decreases rather than increases the need for IP law.

But here too inertia and politics matter. The IP system has served us (reasonably) well for a long time by creating artificial scarcity. And a lot of people stand to benefit from that system. Gradually reorienting creation away from scarcity and toward abundance requires an openness to innovation without IP (Lemley, 2015).

#### Luxury goods and artificial scarcity

One might dismiss the regulatory and IP examples above as evidence of flaws in a political and economic system. Surely, they would argue, the market itself would embrace abundance if left free to do so. Nonetheless, there is some reason to believe that the market responds to abundance by creating artificial scarcity. Societies have long had “sumptuary codes”—rules that distinguish the privileged from the masses by forbidding the masses from owning or displaying certain types of things (Beebe, 2010; Bechtold and Sprigman, 2022). Conspicuous consumption is an effort to flaunt wealth by displaying an excess of things that are scarce in the world at large.

That instinct may persist in society and in the law even in the face of abundance. As Deven Desai has shown, in fact the logic of branding is to create an artificial difference especially when a good is a commodity that is often quite abundant. A close look at the history around the Industrial Revolution with its increased production of *competing* and sometimes over-supplied commodity goods, better transportation, and the desire and ability of producers to reach consumers directly, led to advertising and branding strategies (Desai and Waller, 2010). These strategies allowed producers to convince customers to ask for a product by name such as Heinz Ketchup (Desai and Waller, 2010). Branding influenced what is on store shelves while also enabling producers to extract as much as “20, 25, or 30 percent price premium for a branded good” (Desai and Waller, 2010). And it even persuaded consumers to pay 70% more for brand-name over the counter drugs than their identical generic counterparts, despite government regulation that ensures that the drugs are the same.^17^

This tactic crosses from goods like wheat over to luxury items. Thus, Barton Beebe has suggested that the point of trademark law's protection of luxury brands is to serve as a modern sumptuary code, allowing the rich to distinguish themselves from the masses by displaying their expensive watches and handbags (Beebe, 2010). Certainly it is hard to understand otherwise why people will pay thousands of dollars for a Gucci bag when a bag of equal quality, often made by the same people, is available for a fraction of the price (Desai, 2012). And the demand for counterfeit luxury goods suggests a desire on the part of the have-nots to participate in the game (or at least be perceived to do so). Fashion trends and fast copying of fashion show similar trends (Raustiala and Sprigman, 2006; Hemphill and Suk, 2009; Greenstein).

The modern phenomenon of NFTs is an even clearer example. NFTs are valuable precisely because they create artificial scarcity around things that are for the most part identical to works digitization has made available to the masses for free. You can own an NFT of the Mona Lisa, but you don't own the Mona Lisa itself, and indeed you don't have any greater access to digital reproductions of the Mona Lisa than the rest of the world does. What you own—*all* you own—is the claim to scarcity. You may be the only one (or one of only a few) who owns an NFT of a particular work of art or video clip. But the only thing you own is the scarcity itself. And the “thing” that is scarce is a precise replica of the very same digital information the rest of the world has access to. Joshua Fairfield's chapter in this volume discusses the role of scarcity in NFTs (Fairfield).

This may say something deep about the desire to compete in human nature, or at least in capitalist society. Perhaps replicating scarcity is innate in people because it gives them something to compete over and therefore a way to measure themselves against others. Or perhaps it is innate in capitalism or our conception of value. It may even be a consequence of the skewed distribution of resources in a world that is moving from scarcity to abundance. A few people have an enormous amount of money, and the things money buys are scarce resources, so they invest their money in those resources even if the scarcity is entirely artificial. They may do so merely because they have the money. But they may also do so to signal that they can. The ability to pay huge sums for an NFT signals status in a social order. It is what Stephanie Bair's chapter in this volume identifies as a “positional good” (Plamondon, 2022).

Whether the world will value any particular artificial scarcity is an open question. As a recent story about an NFT for Jack Dorsey's first tweet shows, one can buy an NFT for $2.9 million, try to sell it for an absurd $48 million, only to find that the most offered at the time is $3,600 (Plunkett, 2022). But the numbers can just as easily go the other way. And the underlying instinct to value that which is rare may be more than a mere artifact of our scarcity-based economics. It may be rooted in our culture or even hard-wired into our brains.

As legal re-creations of scarcity go, NFTs seem somewhat less harmful to society than overly strict IP laws or other efforts to fight abundance. They do not, after all, deprive others of access to the thing that is being made artificially scarce. We can all wear purple, and we can all have access to the Mona Lisa in digital form. Their most harmful effect is likely the energy consumption required to trade them from person to person.

But perhaps we should be troubled by the instinct to distinguish haves from have nots, even if the distinction seems entirely artificial. If people are generally happier in more egalitarian societies, the instinct to declare a few winners (and by implication, lots of losers) may be harmful in itself. We turn to the distributional consequences of abundance in the final section.

### Labor, capital, and distributing abundance

While getting things for free (or close to it) seems like a boon to the economy, a number of commentators worry that salaries of most people in the country are based on jobs performing tasks that may soon be obsolete.^18^ If technology delivers our goods for us without trucks or stores, 3D printers manufacture our goods, gene assemblers take over a growing share of our health care and agribusiness, and robots provide many basic services, what is left for people to do?^19^ They could create the things machines will produce and deliver, but as the growth of the gig economy demonstrates, that creation may not be accompanied by a healthy paycheck. Just as happened with farming, our productivity will continue to increase, but it will be machines, not people,^20^ that generate that additional productivity (Rifkin, 1996; Rotman, 2013). Hora's chapter in this volume discusses the role of “servitization” in accelerating this trend across multiple computer industries.

If the returns to productivity accordingly accrue to capital, not labor, the result may be to deepen income inequality (Piketty, 2014). Some worry about massive unemployment, the decline of the middle-class professional, and exacerbating the growing gap between rich and poor (Autor and Dorn, 2013; Evans, 2014). And there will certainly be disruptions in economic structures that we have built around office work and middle-class roles. Mehra, for instance, notes that we have built much modern infrastructure around the assumption that people will travel to offices to work, but the pandemic—and the communications technologies it showcased—may mean that will no longer be true.

To the extent that our economy is based on an ever-expanding spiral of consumption, a long-term drop in the cost of most goods could trigger a fundamental economic contraction or social unrest. Work is central to human social identity, and in the past those displaced by technology have reacted violently against it (Friedman, 2014). More recently, despite the almost 40 year run of low inflation and low-cost goods that post-Soviet globalization created, almost all of that growth has accrued to the benefit of the rich rather than the middle class. Frustrations about wages and income inequality ironically generated a backlash that helped launch Donald J. Trump into the White House—and therefore make those problems worse.

One might also worry about vesting more and more power in the companies that control the networks over which information flows, companies that face little competition and seem increasingly less likely to be subject to common-carrier regulation (Werbach, 2014). And other aspects of our legal system, like torts, will have to change when the people who produce goods are no longer large companies who design them, but rather the very individuals who might be injured by them.^21^ These near-term issues are real, but more important they point to a larger pattern underlying the hopes and fears about abundance.

The ride-sharing industry presents a good example of how technology can both improve people's lives by eliminating scarcity and still create complex dynamics based on who benefits. People had free-time and cars that sat idle. Thanks to software and the Internet, Uber and Lyft connected drivers with riders. Add in GPS available to anyone with a smart phone and the world of licensed taxi drivers who knew roads and needed to be booked with dispatchers went away.

For users, this was unquestionably a good thing. Millions of people had access to effective point to point transportation in a way they never had before. For drivers, the situation was more complicated. Taxi drivers lost out, because they had built a lucrative business based on artificial scarcity imposed by taxi commissions that regulated entry and prevented price competition (Lemley and McKenna, 2020).

What about ride-sharing drivers? On the one hand, more people had side jobs or even fulltime jobs driving people around. The core technology allowed people not only to drive people places but also run errands and deliver goods. And work flexibility is a godsend for many people who need to supplement their income but have family obligations that don't allow them to take a full-time job. On the other hand, concerns about pay, job benefits such as health care, and more surfaced. Cities and states have experimented with regulations and even some nascent movements to unionize have emerged.

While these issues are resolved, the underlying technologies of abundance may make the debates less acute if not irrelevant. For the steady improvement of autonomous vehicles and delivery systems points to a world where machines are the main workers as it were and a fewer humans run the system. Thus, a new abundance cycle will begin with plentiful and hopefully greener, safer, and more efficient transportation. That shift, however, displaces drivers and errand runners who will need new work. Solving these challenges is where government and social policy enter the picture.

One way to frame the problem is to ask “Does technology-driven abundance foster a system where a few at the top live off the surplus created by the many at the bottom who have “only a bare subsistence”? (Graeber and Wengrow, 2021). As we have suggested, technologies of abundance open up the possibility space, making it possible to get more food, more shelter, and more consumer goods to more people more cheaply. But if all they do is reduce the cost of those things in an economic structure that is still driven by scarcity, whether or not people benefit from that abundance depends on whether their income goes away as well (and whether governments will step in to provide access to cheap necessities to those who no longer have the income to pay for them). Indeed, the shift from labor to capital returns the technologies bring could accelerate the “hollowing out” of the middle class in our current economic system (Petersen, 2020). It becomes critical to think not just about how abundant things are, but about whether and how people have access to those things. Arewa's chapter in this volume suggests we have done a poor job so far of ensuring that everyone has access to technologies of abundance.

By one account “An average 61% [of people worldwide] believe that their current positions will be greatly affected by technology change or globalization” (Kovacs-Ondrejkovic et al., 2019). While these risks are substantial, there are reasons for optimism. This is not the first time technology or market forces have fundamentally disrupted our economy. We were alive when the United States was considered a leader in manufacturing, and making products employed a substantial share of our workforce.^22^ And we're not that old. Today only 10 percent of our jobs come from manufacturing; the rest have been sent overseas or replaced by automation (Rotman, 2013). The loss of manufacturing jobs created substantial disruption, but it did not destroy our economy or lead to a long-term increase in unemployment. Rather, it created transition issues for individual workers, but the workforce as a whole transitioned into service and technology jobs.^23^ Even industries still in transition because of digitization and the Internet, bring new opportunities along with disruption.^24^

Abundance technologies promise the same sorts of improvements, reducing the cost of material things, health care, and services and greatly expanding their availability (Diamandis and Kotler, 2012; Cowen, 2013). They may even provide those benefits while reducing the environmental footprint of consumption: the small bit of electricity it costs to download a song does far less harm to the world than manufacturing plastic disks, putting them in plastic cases, trucking them to retail stores, and having people drive to the stores to buy and sell them (Rifkin, 2014). 3D printing and robotics may offer similar environmental benefits.

Asking what we will do in a world where no one has to work helps unpack what steps might be needed to address the social shifts abundance fosters. Even if no one had to work to survive, it seems unlikely that people would do nothing. Humans seems to thrive when they are productive. Maybe they will come up with new creative endeavors, making art or writing the great American novel. Maybe they will plow the benefits of abundance back into the capital economy, continuing to work hard in order to buy more and better things or even more artificially scarce things like NFTs and luxury handbags. Either way, John Maynard Keynes' 1932 dream that increases in productivity would mean that people would only work 15 h a week, because there would simply be no need to work more than that to pay for necessities, is unlikely for now (Keynes, 2010). But as automation, robotics, and artificial intelligence develop, that future may be closer than it seems today.

How society reacts to new technologies of abundance depends critically on how the gains from that abundance are distributed. In the last 40 years, essentially all the returns from technology and productivity have gone to capital, not labor. And because capital was the province of the rich, that meant that those gains have exacerbated rather than reduced income inequality. The U.S. tax system worsens the problem by favoring corporations over individuals and capital over labor productivity. It is important to ensure that everyone benefits from abundance. One way to do that is to reverse our decades-long emphasis on capital at the expense of labor, adopting tax and economic policies that favor people over corporations, or at the very least treat them equally. No less than Microsoft founder Bill Gates has called for a robot tax to slow the effects of automation and fund other employment (Delaney, 2017). Another is to adopt the principle of Equal Relative Abundance, Kop suggests in his contribution to this volume, supporting technologies of abundance only to the extent they grow the pie for everyone.

Even if technology-driven abundance continues to reward capital and not labor, society has options. A recent idea has been to embrace some type of universal basic income (UBI). The notion of UBI has been around for at least two centuries (Van Parijs, 2014; Bidadanure, 2019).^25^ Thinkers such as Thomas Paine, the Belgian socialist Joseph Charlier, John Stuart Mill, James Meade, Martin Luther King, Jr., James Boggs, Milton Freidman, and feminists who were part of “the Wages for Housework movement in the 1970s” have proposed variations on the idea (Bidadanure, 2019). Alaska, the Eastern Band of Cherokee Indians in North Carolina, Canada, Brazil, Finland, Germany, Spain, The Netherlands, Iran, Kenya, Namibia, India, China, and Japan have all tried some form of UBI (Samuel, 2020). The idea has gained renewed interest in the U.S. because of “[t]he growth of income and wealth inequalities, the precariousness of labor, and the persistence of abject poverty” (Bidadanure, 2019). But another driver “is without a doubt the fear that automation may displace workers from the labor market at unprecedented rates that primarily explains the revival of the policy, including by many in or around Silicon Valley” (Marinescu, 2019). Although the details of such ideas and their feasibility is well beyond the scope of this essay, we note that several UBI experiments comport with one of our intuitions: that freedom to do what one wants does not lead to less work (Samuel, 2020). Instead, when UBI has been tried, “baseline educational and health outcomes [often improved] especially among the most disadvantaged]” with little “negative effect on work” (Marinescu, 2019). By extension, if abundance technologies mean we need less labor and UBI can cover basic needs, people are likely to be happier, take part time jobs they like, and freer to pursue work they wish to do, rather than have to do (Van Parijs, 2014).

Increased taxes on capital (like Bill Gates's robot tax) might be used to fund a UBI. Or the funds might allow the U.S. to borrow from the Danish Flexicurity program where employees sign up and pay for 2 years of unemployment insurance, and the government runs education and retraining programs (Working in Denmark). Indeed, no less than the World Economic Forum has embraced the idea of the Reskilling Revolution (World Economic Forum, 2019; Denmark, 2022). The Danish and WEF approach of public-private partnerships to reskill workers as abundance technologies continue to disrupt puts the correct emphasis on how to evolve with technology rather than blaming it for our woes. As Peter Hummelgaard, Minister for Employment, Ministry of Employment of Denmark, has offered, “When the weather forecast says a hurricane is coming, we act. We take precautions for our own homes. We help our neighbors and we join our efforts in local communities. We take joint responsibility because we are aware of the dire consequences if we do not act” (Hummelgaard, 2020). Funding programs to allow the U.S. workforce to reskill or upskill is a sound strategy that the U.S. should pursue so that the wealth generated by technologies of abundance can have a better chance of reaching more people.

Retraining for a world of abundance, though, will not necessarily occur fully within the framework of a scarcity-based economics driven by physical things sold for a price. While one possible future involves recreating scarcity, either by developing new goods that are scarce or by artificially duplicating it with brands, that is not the only possible path. The economy we have known for over a century may play a smaller and smaller role in defining how people actually live their lives. As Jeremy Rifkin puts it.

As more and more of the goods and services that make up the economic life of society edge toward near zero marginal cost and become almost free, the capitalist market will continue to shrink into more narrow niches where profit-making enterprises survive only at the edges of the economy... We have been so convinced of the economics of scarcity that we can hardly believe that an economy of abundance is possible. But it is Rifkin (2014).

We may spend more of our time inventing and creating, not because we are paid to do so but simply because we have that time to spend.^26^ Post-scarcity technologies give more of us the means to be more creative. They give us an abundant source of raw materials to play with, mix, and remix (Lessig, 2008). They free us from constraints that demand our time and our attention (Mullainathan and Shafir, 2013; Heck et al., 2014). That creates room for great optimism about the future—but only if we can adapt our economic system to ensure that we benefit from the technologies of abundance.

## Conclusion

Our hope is that with better technology, we can create abundance while *not* falling into old patterns of haves and have nots. Such a future may appear to be a *Star Trek* one, at least a *Star Trek the Next Generation* one, where everything is abundant and money no longer exists. That future is far, far away. Yet, perhaps replicators are not as far off as it seems. For things such as music or movies that can be fully digitized for creation and distribution and we are closer to a replicator world than not. Advances in artificial intelligence mean that systems can now generate new writings, pictures, and even movies after being given some data and instructions. Thus, the world where we might say, “Computer. Image. My House, Starry Night style,” and a fantastic digital (or 3D-printed) image is ready in minutes is essentially here.^27^ Of course, the canvas and paints are physical, and energy is still not magically at Star Trek almost zero-costs. And we cannot yet digitize physical things to transport them or take raw energy and reorder it into matter. Nonetheless, advances in the production of energy, food, media, goods, services, and more have brought a wave of abundance not seen since the industrial revolution. The advances have, however, also coincided with new winners and new levels of inequality, as well as efforts to reconstruct the scarcity on which our traditional notion of economics depends. We do not claim to solve the overall tension in this essay or collection. But we think the essays in this book offer important ruminations on the nature of technology-driven abundance, its effect on how we organize society, and the way it might lead us to a better future.

## Author contributions

Both authors contributed equally to the research and writing of this paper. Both authors contributed to the article and approved the submitted version.

## Footnotes

^1^See, e.g., DeLong and Summers (2001) (“[T]he most basic condition for economic efficiency [is] that price equal marginal cost.”); Desai (2012) (describing how branding practices allow a firm to move beyond the 4Ps of product, price, place, and promotion and charge and charge above marginal costs).

^2^For a detailed critique and engagement with the nature of property rights and systems supporting them, see, Frischmann and Ramello, Ghosh.

^3^Shubha Ghosh offers an insightful exploration of the problems with the relationship among public goods, natural monopoly, and intellectual property policy (Ghosh, 2008).

^4^“Copies of books sold more than doubled from one billion in 1993 to 2.3 billion in 2007. The number of titles produced increased to more than 70,000 in 2002 and to almost 300,000 in 2012.” When we factor in self-published and print-on-demand books, that number rose to “more than three million in 2010” (Travis, 2015, p. 8).

^5^Local Motors pivoted, however, from passenger cars to autonomous shuttles and that business choice did not work. But that doesn't mean that the idea itself failed (Voulpiotis, 2019; Ballen, 2022).

^6^Onshoring in the fullest sense of bringing most manufacturing back to the U.S. is in the future is unlikely. What seems to be happening is some sectors are seeing whether they can leverage new technologies and be competitive with operations in the U.S. (Smialek and Swanson, 2022). Most of the changes are moving away from China to other countries such as Vietnam and Mexico—a concept some call reshoring—as a way to improve supply chain reliability while still having low-costs to produce (Smialek and Swanson, 2022).

^7^On the dynamics of attribution, IP, and information rich environments, see, Desai (2011).

^8^For example, Eric von Hippel notes the willingness of user innovators to give their ideas away calls into question the basic theory of IP (Von Hippel, 2005).

^9^As Desai and Magliocca argue “[F]irms would be better off embracing this change in production to cultivate new markets instead of trying to win Pyrrhic victories in Congress and the courts” (Desai and Magliocca, 2014).

^10^As we discuss below, the critique that abundance may fuel new consumption has some merit; and yet is simplistic especially when the critique focuses on technology rather than social forces around the implementation and effects of the technology.

^11^As Rifkin notes, “In 1850, 60 percent of the working population were employed in agriculture.” (Rifkin, 2014).

^12^According to the 2021 the U.N.'s Statistical Yearbook World and Food Agriculture “The world average dietary energy supply (DES), measured as calories per capita per day, has been increasing steadily to 2,950 kcal per person per day over the period from 2018 to 2020, up 9 percent compared with 2000 to 2002” (FAO, 2021).

^13^As David Beasley, head of the United Nations World Food Programme noted spikes in food prices and supply lead to protests and “both the war in Syria and the Arab Spring uprising in 2011 were preceded by food price spikes and supply issues” (Egan, 2022).

^14^As one review of data from the U.S. Energy Information Administration between 2010 and 2019 found, “In terms of total electricity produced from renewables, California (97 million MWh), Texas (91 million MWh), and Washington (74 million MWh) are the national leaders” (Heacock, 2022).

^15^Similarly, some states try to slow of block solar power deployment in the first place because of the demands of the dominant power company in their state.

^16^Nonetheless old habits take some time to die out as shown by Honda's cease and desist letter campaign regarding plans that allow 3D printing of Honda parts (Stumpf, 2022).

^17^One study noted price disparities of up to 80% in over-the-counter drugs (Aufegger et al., 2021). There is good literature on the role of advertising in persuading vs. informing consumers (Brown, 1948; Lemley, 1999; Beebe, 2004; Desai, 2012).

^18^The number of people talking about this has gone from essentially zero a few years ago to legion today (Brynjolfsson and McAfee, 2011; Autor and Dorn, 2013; Rotman, 2013; Evans, 2014).

^19^The Gartner Group estimated in 2014 that one in three of today's jobs will be performed by machines in 2025 (Thibodeau, 2014).

^20^As one study noted, farms grew and used mechanized production; thus, labor numbers went down with farm households seeking “off-farm income/work” because of time to do so and the need to do so to move farm households above the poverty line (Dimitri et al., 2005).

^21^Law responds to risk either by regulating entry or by regulating consequences. Tort law has generally regulated consequences, but that seems less and less feasible in a world in which production is noncommercial and democratized (Engstrom, 2013; Desai, 2014). Entry regulation seems likely to be both ineffective and a bad idea even if it could work (Desai, 2014). Thus, we may need to replace tort law with a social safety net as it becomes harder and harder to find those who make unsafe products and hold them liable.

^22^Manufacturing represented thirty percent of all U.S. jobs in the 1950s and 1960s (Rotman, 2013).

^23^As Rotman has said, “[N]o historical pattern shows these shifts leading to a net decrease in jobs over an extended period.... ‘[W]e have never run out of jobs. There is no long-term trend of eliminating work for people”' (Rotman, 2013).

^24^A study by the McKinsey consulting group, for instance, found that the Internet has created nearly three times as many jobs as it has destroyed (Pélissié du Rausas et al., 2011). Another study indicates that as of 2021, the commercial internet economy accounts for more than 17 million jobs as compared to 2008 (Deighton and Kornfeld, 2021). The exact “quality and composition” of the new jobs is, however, a subject still under study (Adams, 2018).

^25^Related ideas connect from further back in history (Basic Income Earth Network, 2022).

^26^Yochai Benkler notes that historically this option has usually been reserved for the wealthy and those who have time on their hands: Children and teenagers, retirees, and very rich individuals can spend most of their lives socializing or volunteering; most other people cannot. … human creative capacity cannot be fully dedicated to nonmarket, nonproprietary production all the time. Someone needs to work for money, at least some of the time, to pay the rent and put food on the table (Benkler, 2006).

^27^As we wrote this essay and posited this idea, Google in fact announced a text-to-image-AI (Vincent, 2022).
